# The Role of Bronchoalveolar Lavage in Therapeutic Antimicrobial Choices for Hematologic Patients with Pulmonary Infiltrates

**DOI:** 10.3390/medicina61010118

**Published:** 2025-01-14

**Authors:** Filippo Patrucco, Mattia Bellan, Davide Martinotti, Giuseppe Ielo, Paola Rebecca Iovine, Martina Mascheroni, Francesco Todisco, Martina Ubaldi, Nadia Castaldo, Francesco Gavelli, Alberto Fantin

**Affiliations:** 1 Division of Respiratory Diseases, Internal Medicine Department, AOU Maggiore della Carità di Novara, 28100 Novara, Italy; 20010557@studenti.uniupo.it (D.M.); giuseppeielo@hotmail.it (G.I.); ipaolarebecca@gmail.com (P.R.I.); m.mascheroni8@studenti.uninsubria.it (M.M.); francesco.todisco@maggioreosp.novara.it (F.T.); ubaldimartina1@gmail.com (M.U.); 2 Emergency Medicine Department, AOU Maggiore della Carità di Novara, 28100 Novara, Italy; mattia.bellan@med.uniupo.it (M.B.); francesco.gavelli@med.uniupo.it (F.G.); 3 Department of Translational Medicine, Università degli Studi del Piemonte Orientale, 28100 Novara, Italy; 4 Department of Pulmonology, S. Maria della Misericordia University Hospital, 33100 Udine, Italy; nadiacastaldo.nc@gmail.com (N.C.); alberto.fantin@asufc.sanita.fvg.it (A.F.); 5 Department of Medicine, Respiratory Medicine Unit, AOU Integrata of Verona, University of Verona, 37100 Verona, Italy

**Keywords:** bronchoalveolar lavage, immunosuppression, hematologic diseases

## Abstract

*Background and Objectives:* Lower respiratory tract infections are particularly frequent in hematological patients; their early diagnosis and the timely start of targeted therapy are essential. Bronchoalveolar lavage (BAL) can provide a microbiological sample from the lower airways in a minimally invasive way. This study aimed to determine the diagnostic yield of BAL in hematological patients for microbiological purposes and its influence on modifying the therapeutic strategy. *Material and Methods:* This multicenter, retrospective, observational study included data from 193 consecutive patients from two centers from January 2020 to October 2022. The patients underwent a bronchoscopy with BAL in cases of pulmonary infiltrates suspicious of pulmonary infection. The demographic data, presenting symptoms, level of immunosuppression, chest CT changes, BAL sampling results, and antimicrobiological drug administration were analyzed. *Results:* Of the 193 procedures, 143 (74.1%) were performed on hospitalized patients, while 50 were performed on outpatients. In 53.9% of the cases, the BAL isolated at least one germ; in particular, if the procedure was carried out within 72 h of presenting symptoms, the probability of isolating the germ increased significantly (74.3%, *p* = 0.04). Among the isolated germs, 59.4% were viruses, 28.6% were bacteria, and 12% were fungi. The patients with higher immunosuppression and the febrile ones underwent BAL earlier than the patients with mild immunosuppression (*p* = 0.01) and those with other presenting symptoms (*p* = 0.0001). BAL positivity led to a change in empirical antimicrobial therapy in 79 out of 104 cases (77% vs. 36.3%; *p* < 0.001); these data were also confirmed among the hospitalized patients (81% vs. 44%; *p* < 0.001). The isolation of a pathogen through BAL and the degree of patient immunosuppression negatively influenced patient survival (*p* < 0.05 and *p* < 0.01, respectively). *Conclusions:* BAL is confirmed as a valid approach for evaluating pulmonary infiltrates in hematological patients, given the excellent clinical impact and high diagnostic yield, mainly if performed early after symptom presentation. However, ongoing antimicrobial treatments at the time of BAL may have potentially affected the diagnostic yield of the procedure.

## 1. Introduction

Patients affected by hematologic malignancies present with an increased susceptibility to severe infections due to the disease itself and to the myelosuppressive and cytotoxic effects of the chemotherapies administered [1,2]. Hematopoietic stem cell transplantation (HSCT) constitutes the gold standard for treating many hematologic malignancies. About 90% of patients are estimated to develop infections during the transient phase of bone marrow aplasia before transplantation [3,4,5].

When symptoms and signs of infection are evident, or elevation of inflammatory markers occurs, empiric therapy is initiated, guided by multiple factors: the severity of immunosuppression and neutropenia, the possible site of infection, signs and symptomatology of the patient, the local epidemiology, any previous infection with multidrug-resistant agents, the existence of specific nosocomial pathogens, previous antibiotic treatment, and the presence of any allergy or toxicity [1,6].

In patients treated for hematologic malignancies and those undergoing HSCT, pulmonary infiltrates are very common: some studies have suggested an incidence between 13% and 60%, which varies depending on the underlying disease and treatment type [7,8,9]. A CT scan of the chest at high resolution (HRCT) is the best method for diagnosing pulmonary infiltrates, particularly concerning bacterial, viral, or fungal pneumonia [10].

Bronchoalveolar lavage is a useful diagnostic tool for directly sampling alveolar content [11,12]. Several studies have shown that in immunosuppressed patients with hematologic malignancies (HMs) or after an HSCT, adjustments to antimicrobial therapy are frequently performed as a result of the invasive microbiological test results [13,14,15,16]. A bronchoscopy with BAL is a common approach to evaluate nodular and diffuse infiltrates in patients with HM and HSCT because of the low risk of complications (such as hemorrhage or pneumothorax), good patient tolerance, and its diagnostic yield, which has been estimated to be from 70% [17] to 90% [18]. The heterogeneity of the patients and their clinical conditions, the time at which the BAL is performed, the empiric antibiotic therapy initiated, and the location of the lesions to be investigated, on the other hand, are confounding factors [19].

The present study aims to define the diagnostic yield of BAL performed in hematologic patients with pulmonary infiltrates suspected to be of infectious origin to evaluate the factors that can influence diagnostic yield and to determine whether its results lead to a change in antimicrobiological strategy.

## 2. Materials and Methods

### 2.1. Study Design

This multicenter, retrospective, observational study included patients with different hematologic diseases who underwent BAL. The indication for performing BAL was for microbiologic pulmonary sampling with the suspicion of infectious pathology of pulmonary origin.

The data collected came from the Respiratory Disease Units of two centers, Novara and Udine. Data were retrieved from data of consecutive patients from 30 January 2020 to 26 October 2022.

Of the initial 206 patients, 13 were excluded because of a lack of data or because they underwent BAL for reasons unrelated to the study’s objective. For each patient, the date of birth, inpatient or outpatient setting, hematologic diagnosis, number of days between the onset of symptoms and the performance of BAL, degree of immunosuppression (not immunosuppressed, mild, moderate, severe immunosuppression according to the count of neutrophils and Nirenberg A. et al.: non-immunosuppressed > 1.51 × 10^3^/μL, mild grade 1.5 to 1 × 10^3^/μL, moderate grade 0.9 to 0.5 × 10^3^/μL, severe grade ≤ 0.49 × 10^3^/μL) [20], presenting respiratory symptoms (cough, fever, or dyspnea), laboratory examinations at first evaluation (complete blood count, c-reactive protein-CRP, serum creatinine, and procalcitonin-PCT), significant alterations at HRCT scan (negative for thickenings, nodular thickenings, consolidations, ground glass opacities, or pleural effusion), hematochemical examinations close to the day when BAL was performed (with the exact measurements listed above), BAL results (bacteria and mycobacteria culture, respiratory and herpes virus film-array panel, mycete search, and galactomannan assay), current anti-microbial therapy at the time of BAL (antibiotic, antiviral, or antifungal), antibiotic and/or antiviral prophylaxis (Trimethoprim–Sulfamethoxazole/Aciclovir–Valacyclovir), any change in therapy post BAL, the discharge rate of hospitalized patients, 30-day survival, and the date of eventual death.

For microbiological analysis, samples were tested for the presence both of herpesviruses (HSV-1, HSV-2, VZV, HCMV, EBV, and HHV-6) using a RealTime PCR (Elite MGB Kits, ELITech, Turin, Italy) and respiratory viruses (influenza A, influenza B, RSV-A, RSV-B, Metapneumovirus, Rhinoviruses, respiratory Enteroviruses, Coronaviruses, human Bocavirus, Adenoviruses, and Parainfluenza 1, 2, 3, 4) with a different RealTime PCR assay (Anyplex RV16, Seegene, Seoul, Republic of Korea). RealTime PCR was used to identify Mycobacterium tuberculosis compelx DNA and galactomannan detection was performed using a double-sandwich ELISA and considered positive when greater than 0.5. Standard bacterial, mycobacterial, and fungal cultures were performed for each sample.

### 2.2. Statistical Analysis

Categorical variables are presented as absolute values and percentages, while continuous ones are presented as the mean ± SD or median and interquartile range [IQR] as appropriate. We used the t-test to test for differences between continuous variables. We used the ANOVA test for comparisons between three or more averages and Tukey’s test for multiple comparisons. Fisher’s exact test was used when appropriate to compare categorical variables. Survival estimates were performed using Kaplan–Meier survival analyses, and the differences between groups were determined using the log-rank test. All *p*-values are considered two-tailed, and significance was set <0.05. MedCalc software (MedCalc Software Ltd., Ostend, Belgium) was used for the analyses.

## 3. Results

### 3.1. Population

A total of 193 patients with hematologic diseases who underwent a bronchoscopy with BAL for suspected pulmonary infection were included in the study, with 98 patients from Novara and 95 from Udine. The demographic characteristics of the patients are detailed in Table 1. The mean age at the time of bronchoscopy was 58.8 years, with the majority of patients aged between 61 and 80 years old. Most of the patients were male (57.1%). Out of the procedures, 74.1% were performed on inpatients, with the remaining 25.9% performed on outpatients. Patients were categorized based on the degree of immunosuppression [20], with the mild grade being the most common (40% of patients) and the moderate grade being the least frequent (8.8% of patients). The most prevalent hematologic diseases was acute myeloid leukemia (37.3%), followed by multiple myeloma (16.1%). Symptoms of suspected pulmonary infection were present in 88.1% of patients, with fever being the most common (75.8%). The average white blood cell count (WBC) at presentation was 6.5 × 10^3^/μL, with a mean absolute neutrophil count of 4.1 × 10^3^/μL. The mean CRP level was 28.3 mg/dL, and PCT was measured in 34 cases with a mean value of 0.3 mg/dL. HRCT showed lung parenchymal alterations in 97.4% of cases, with ground-glass opacities (35.8%) and consolidations (29.9%) being the most frequent. Among hospitalized patients, the discharge rate was 83.9%, with a 30-day mortality rate of 9.7% after symptom onset and 17.1% after bronchoscopy with BAL.

### 3.2. BAL Timing

**Figure 1:**
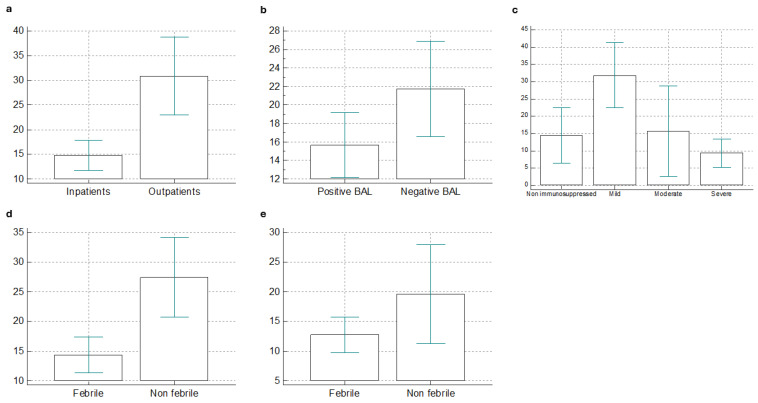
Days elapsed from respiratory symptom onset and bronchoscopy in different subgroups of patients: (**a**) inpatients vs. outpatients; (**b**) positive vs. negative BAL; (**c**) non-immunosuppressed vs. different severity of immunosuppression; (**d**) febrile vs. non febrile all patients; (**e**) febrile vs. non febrile only inpatients.

The mean time from the onset of respiratory symptoms to the performance of BAL was 18.5 days, with inpatients undergoing the procedure earlier than outpatients. Among inpatients, the mean time was 8.4 ± 10.8 days, while among outpatients, it was 30.3 ± 25.5 days (*p* < 0.001) (Figure 1a).

For all patients, those who had at least one isolation at BAL underwent the sampling procedure earlier (15.7 ± 17.9 days vs. 21.8 ± 24.1 days, *p* = 0.05), as shown in Figure 1b. This difference was not significant when considering only hospitalized patients (13.5 ± 16.3 days in cases of positive BAL vs. 16.8 ± 20.7 days with negative BAL, *p* = 0.29). Among hospitalized patients, performing the BAL early after symptom onset (within 72 h) was associated with a higher probability of identifying a germ (74.3% vs. 53.7%, *p* = 0.04). Additionally, there was a significant correlation between the degree of immunosuppression and the time from symptom onset to BAL execution among hospitalized patients (*p* < 0.001): patients with severe immunosuppression underwent bronchoscopy earlier (8.4 ± 7.3 days), followed by non-immunosuppressed patients (10.5 ± 14.4 days), patients with moderate immunosuppression (11.5 ± 23.8 days) and patients with mild immunosuppression (11.5 ± 19.5 days).

Non-immunosuppressed patients had BAL performed earlier than those with mild immunosuppression (10.5 vs. 23.3 days, *p* = 0.03), while patients with mild immunosuppression underwent the procedure later than those with severe immunosuppression (23.3 vs. 8.4 days, *p* = 0.01) (Figure 1c).

There were significant correlations between admission blood test results (lymphocyte counts, hemoglobin concentration, CRP, and creatinine levels) and the time from symptom onset to BAL performance, as shown in Table 2. When considering the entire population, febrile patients underwent BAL earlier (mean 14.3 ± 17.1 days) than the afebrile ones (27.5 ± 25.9 days) (*p* = 0.0001) (Figure 1d). However, this difference was not observed when considering only inpatients: 13.6 ± 17.2 days in febrile patients and 19.6 ± 21.5 days in afebrile patients (*p* = 0.121) (Figure 1e).

### 3.3. Microbiological Findings

Out of the 193 bronchoscopies with BAL performed, 104 were positive for at least one pathogen (53.8%), while the remaining 89 (46.2%) were sterile. Among the 192 isolated germs, viruses were the most common (59.4%), followed by bacteria (28.6%) and fungi (12%). Human Herpesvirus 6 (HHV-6) was the most frequently isolated virus, occurring 30 times (26.3% of viruses, 15.6% of the total); *Pseudomonas aeruginosa* was isolated 9 times (16.3% of bacteria, 4.7% of the total); *Pneumocistis jirovecii* was the most common fungus, isolated 10 times (43% of fungi, 5.2% of the total) (Table 3).

Inpatients had a higher rate of positivity for at least one pathogen (84/143, 58.7%) compared to outpatients (19/50, 38.0%, *p* = 0.01). Patients with germ isolation on BAL were more likely to have a change in antimicrobial therapy (57/74, 77.0% vs. 28/77, 36.3%, *p* < 0.001); this trend was also seen in the inpatient population (47/58 81% vs. 22/50 44%, *p* < 0.001). The presence of current antibiotic therapy at the time of BAL did not impact the likelihood of isolating at least one bacterium, both among inpatients (26/122 21.3%, vs. 6/20 30.0%, *p* = 0.394) and the entire population (31/138 22.4% vs. 14/52 26.9%, *p* = 0.567). Similarly, antiviral therapy during BAL did not significantly affect the chance of isolating a virus, in hospitalized patients (11/22 50% vs. 48/120 40%, *p* = 0.481) or in the overall population (11/26 42.3% vs. 54/163 33.1%, *p* = 0.379). The same was observed for antifungal therapy, among inpatients (7/73 9.6% vs. 12/69 17.4%, *p* = 0.462) and the total population (9/88 12.2% vs. 14/102 13.7%, *p* = 0.509). The majority of patients were on antimicrobial therapy at the time of BAL with antibiotics being the most common (72.6%), followed by antifungals (46.3%) and antivirals (14.2%). Additionally, 31.3% were taking at least two antimicrobial drugs and nearly 20% were taking four antimicrobial drugs. Out of 190 patients with available data, 150 were receiving both antibacterial and antiviral prophylaxis with Trimethoprim–Sulfamethoxazole being the most common antibiotic prophylaxis in combination with Acyclovir (86/137, 62.7%). Antivirals used alone were less common with only 13 out of 150 patients following such a regimen (8.7%).

There was no association found between alterations in CT scan results (consolidation and ground-glass opacities and nodules) and germ positivity.

### 3.4. Survival

In the general population, 9.3% of patients died within 30 days of BAL, while 16.6% died within 90 days. In contrast, 5.3% died within the first month from symptom onset, and 14.4% died within 90 days.

**Figure 2:**
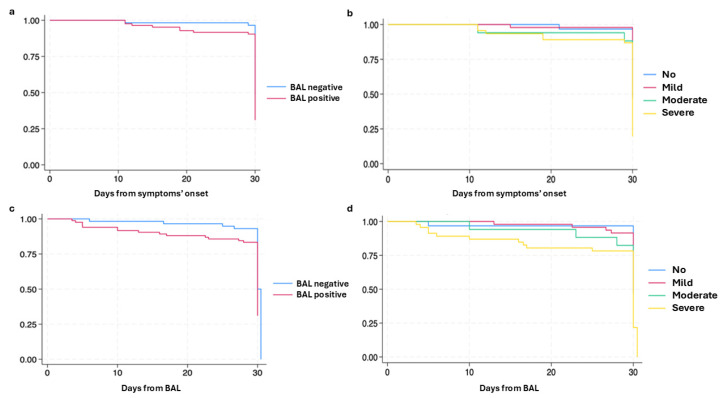
Survival at 30 days from symptom onset and BAL: (**a**) Days from symptom onset: positive vs. negative BAL; (**b**) Days from symptom onset: non-immunosuppressed vs. different severity of immunosuppression; (**c**) Days from BAL: positive vs. negative BAL; (**d**) Days from BAL: non-immunosuppressed vs. different severity of immunosuppression.

Survival data at 30 days from symptom onset and 30 days from BAL were analyzed among inpatients only. Survival at 30 days from symptom onset: patients with positive BAL had a significantly lower survival rate at 30 days compared to those with sterile BAL (*p* = 0.023) (Figure 2a); higher mortality was significantly associated with more severe immunosuppression (*p* = 0.007) (Figure 2b)Survival at 30 days after BAL: mortality was higher in patients with positive BAL (*p* = 0.012) and correlated with the severity of immunosuppression (*p* < 0.001) (Figure 2c,d).

There was no significant difference in mortality based on the types of pathogens isolated (Table 4). Changing antibiotic therapy was associated with a significant increase in mortality 30 days after FBS with BAL (*p* = 0.036) but not since symptom onset (*p* = 0.089). Early BAL within three days of symptom onset did not improve mortality at 30 days after BAL (*p* = 0.71) or at 30 days after symptom onset (*p* = 0.98).

## 4. Discussion

Patients with hematologic malignancies often have weakened immune systems and are prone to developing lung infiltrates from various causes [21]. It is crucial to accurately diagnose the underlying cause to determine the best treatment approach and timing. HRCT is a key diagnostic tool, but it may not always provide a definitive diagnosis [22]. BAL is frequently performed in these patients to gather detailed microbiological and cytomorphologic information, which can guide precise treatment decisions or adjustments to empiric therapy [23]. When hematologic patients present with pulmonary infiltrates, initial empiric therapy is typically based on international guidelines or local protocols [24]. However, the routine use of BAL has been a topic of debate due to advancements in CT imaging and noninvasive microbiological tests that may reduce the need for invasive procedures [19]. Some studies suggest that BAL results may not always significantly impact patient management [22]. This study aims to assess the effectiveness of BAL in identifying infectious pathogens and its potential therapeutic and prognostic implications in hematologic patients with pulmonary infiltrates. Our study found that BAL had a significant diagnostic impact in our patient group, with a germ identified in 53.9% of cases. The rates of germ isolation on BAL in the literature vary widely, ranging from 39% to 65% [22,25]. Zak et al. [26] also found similar prevalence rates of germ isolation in both the intensive care unit (ICU) and endoscopy suite, with the only notable difference being a higher incidence of *Aspergillus* spp. among ICU patients (22% vs. 9%).

The average time between the onset of respiratory symptoms and BAL was 18.5 days. Inpatients tended to have the procedure performed sooner than outpatients, with a mean of 8.4 days compared to 30.3 days. Studies suggest that BAL is most effective when performed within 4 days of symptom onset, especially within 24 h [15]. Our study supported this finding, showing that early BAL (within 72 h of symptom onset) led to a higher likelihood of identifying a pathogen, both in the overall population and in hospitalized patients specifically (74.3% vs. 53.7%, *p* = 0.04). Factors such as logistical challenges, delayed response to antibiotics, and patient clinical status can influence the timing of bronchoscopy. These factors may impact the decision to perform the procedure and the patient’s prognosis.

Among hospitalized patients, the level of immunosuppression affected the timing of the procedure. There was a significant correlation between the onset of respiratory symptoms and the date of bronchoscopy with BAL (*p* < 0.001). Patients with severe immunosuppression underwent bronchoscopy sooner, increasing the chances of receiving targeted therapy earlier. Conversely, patients with mild immunosuppression underwent bronchoscopy later than those with severe immunosuppression (*p* = 0.01). This is a novel finding in the literature and may be due to a bias in case selection: patients with severe immunosuppression were likely monitored more closely (as they were hospitalized) and had earlier access to diagnostic tests. Additionally, the presence of fever influenced the timing of bronchoscopy; febrile patients underwent BAL earlier than non-febrile patients (14.3 vs. 27.5 days; *p* = 0.0001), consistent with previous studies [27].

There are limited studies on the timing of performing BAL in hematologic patients. In our study, BAL was performed on average 8.4 days after the onset of respiratory symptoms in hospitalized patients, which is slightly longer than what has been reported in the literature. For example, in a study by Marchesi et al. [28], BAL was performed around 4 days after identifying a pulmonary infiltrate, showing that early BAL can improve patient survival. Another study by Thorat et al. [29] found that BAL was performed around 6 days after patient presentation, with a trend towards better treatment response, although not statistically significant [30].

It is important to note that the procedures in our study were carried out exclusively in the Division of Interventional Pneumology and did not include procedures performed in the ICU. This may have resulted in an overestimation of the number of days, as patients with severe clinical and respiratory conditions who underwent BAL during their ICU stay were not included.

Overall, a higher rate of positivity to at least one pathogen was observed in BALs performed in inpatients compared to those performed in an outpatient setting (58.7% vs. 38.0%; *p* = 0.01).

Microbiological isolations revealed a total of 192 isolated germs, with viruses accounting for 59.4%, bacteria accounting for 28.6%, and fungi accounting for 12%. HHV-6 was the most common virus, *Pseudomonas aeruginosa* was prevalent among bacteria, and *P. jirovecii* was the most frequent fungus. These findings contrast with the existing literature, which typically indicates a higher prevalence of bacteria over viruses. In a previous study, bacteria were isolated in 33.7% of cases, fungi were isolated in 18.7%, and viruses were isolated in only 2%: *Staphylococcus aureus*, *Aspergillus* spp., and CMV were the most common pathogens [16]. Different studies have reported varying proportions of bacteria, fungi, and viruses, with MRSA, *P. aeruginosa*, *P. jirovecii*, and CMV being frequently identified [23,29]. While some viruses can be definitively diagnosed through molecular analysis in BAL samples, the pathogenic role of certain viruses remains debated, as they may not always cause infection or require specific treatment [31,32].

In our case series, HHV6 was found to be a harmless bystander and not considered pathogenic. On the other hand, EBV and CMV were the most frequently isolated potentially pathogenic viruses, accounting for 14.1% and 13% of total isolations, respectively. The lower incidence of bacterial isolations compared to viral or fungal ones in our study can be explained by the use of broad-spectrum antibiotics given to almost two-thirds of the patients at the time of BAL. This highlights the importance of performing BAL early, before starting empiric therapy, for better diagnostic accuracy [22].

In our study, BAL positivity led to a change in antimicrobial therapy in 76% of cases, with 77% experiencing a more significant change when a germ was isolated upon BAL compared to 36.3% without isolation (*p* < 0.001). This impact was particularly evident in the inpatient population (81% vs. 44%; *p* < 0.001). Compared to other studies, our findings show a higher rate of therapy changes (76%) compared to Cordani et al. (56%) and Hohenadel et al. (84%) [24,25]. Marchesi et al. demonstrated that BAL-guided therapy improved outcomes in terms of pulmonary infiltrates and survival [28]. Interestingly, ongoing antimicrobial therapy did not affect the ability to isolate microorganisms, with 53% of cases yielding positive results despite broad-spectrum treatment. This is consistent with previous studies that also found successful pathogen isolation during antimicrobial therapy [16,30]. Additionally, a high proportion of patients in our study received antibacterial and/or antiviral prophylaxis (79.4%), highlighting the complexity of managing infections in this population [16,30].

The high diagnostic yield of BAL despite multiple lines of antimicrobial treatment may be due to high colonization of pathogenic organisms in the lower airways of immunocompromised patients or increasing antibiotic resistance [26,33].

Despite positive BAL results, therapy changes were not always made because the isolated pathogen was sensitive to current treatment or because there was a lack of more effective or specific therapy, such as for Rhinovirus [26,27].

The discharge rate among hospitalized patients was 83.9%. The mortality rate was 9.3% within 30 days and 16.6% within 90 days from BAL, and 5.3% within 30 days and 14.4% within 90 days from symptom onset. Among the BAL-positive population, the mortality rate at 30 days after symptom onset was 9.5%, while the mortality rate at 30 days after BAL was 16.7%. These findings are partially supported by the literature, with a study showing a mortality rate of 8% at 30 days after BAL in cases with a positive result and a better prognosis for patients with a positive BAL finding [34]. The degree of immunosuppression significantly influenced mortality at 30 days, with patients with severe immunosuppression having mortality rates of 13% and 21.7% when considering symptom onset and BAL performance, respectively. Higher levels of immunosuppression increase the risk of mortality, especially when dealing with more virulent pathogens [30,35].

The change in antibiotic therapy resulted in a higher 30-day mortality rate after BAL at 16.1% but did not impact mortality at 30 days after symptom onset. This contrasts with a study by Marchesi et al. [28] that showed improved prognosis at 120 days when antimicrobial therapy was changed after detecting pulmonary infiltrates. Our study suggests that patients with multiple antibiotic treatments may have more drug-resistant pathogens, leading to the need for therapy changes [25,36]. This change can improve lung disease outcomes but may also indicate previous ineffective treatment or the presence of a co-infection, such as a fungal infection [37].

This study has some limitations. Firstly, its retrospective nature prevents definitive conclusions; however, the results align with previous literature results. Secondly, the inclusion of both inpatients and outpatients with varying disease severity led to different timelines, patient management, and treatment indications. The two cohorts (Novara and Udine) were similar in size and characteristics but may have had different protocols and internal management. Intensive care unit bronchoscopies, often performed on more critically ill patients, were excluded. The use of multiple antibiotics and antifungal and antiviral drugs may have influenced the procedure outcomes, as evidenced by sterile BALs despite symptoms. It is important to note that BAL in hematologic patients is generally safe with low complication rates. Adhering to guidelines and timing the procedure appropriately can provide valuable microbiologic information that may benefit patient outcomes.

## 5. Conclusions

In conclusion, this study confirms that BAL remains a valuable method for assessing pulmonary infiltrates in hematologic patients. It has a high diagnostic yield and clinical significance. Conducting BAL early, within 72 h of symptom onset, enables prompt identification of the causative pathogen, guiding appropriate antimicrobial treatment and reducing the risk of treatment-related side effects.

## Figures and Tables

**Table 1:** Patients’ characteristics.

	N (%)	
Number of patients	193 (100%)	
Centers		
- Novara	98 (50.8%)	
- Udine	95 (49.2%)	
Sex		
- Males	56 (57.1%)	
- Females	42 (42.9%)	
Age (mean ± SD), in years	58.8 ± 14.3	
Inpatients	143 (74.1%)	
Outpatients	50 (35.9%)	
Hematologic diagnosis	N/total population	N/group (%)
	72/193 (37.3%)	
- Acute Myeloid Leukemia (AML)	52/193 (27%)	
- Lymphoma	28/193 (14.6%)	28/52 (53.8%)
- Hodgkin lymphoma (HL)	13/193 (6.7%)	13/52 (25%)
- Non-Hodgkin lymphoma (NHL)	9/193 (4.7%)	9/52 (17.4%)
- Diffuse large B cell lymphoma (DLBCL)	1/193 (0.5%)	1/52 (1.9%)
- Mycosis fungoides (MF)	1/193 (0.5%)	1/52 (1.9%)
- MALTomas	31/193 (16.1%)	
- Multiple Myeloma (MM)	30/193 (15.5%)	
- Other leukemia	12/193 (6.2%)	12/30 (40%)
- Acute lymphocytic leukemia (ALL)	8/193 (4.1%)	8/30 (26.7%)
- Chronic lymphocytic leukemia (CLL)	3/193 (1.6%)	3/30 (10%)
- Chronic myelomonocytic leukemia (CMML)	3/193 (1.6%)	3/30 (10%)
- Chronic myeloid leukemia (CML)	2/193 (1%)	2/30 (6.7%)
- Hairy cells leukemia (HCL)	1/193 (0.5%)	1/30 (3.3%)
- Acute promyelocytic leukemia (APL)	1/193 (0.5%)	1/30 (3.3%)
- Plasma cell leukemia (PCL)	8/193 (4.1%)	
- Other hematologic disorders	3/193 (1.6%)	3/8 (37.5%)
- Myelodysplastic syndromes	2/193 (1%)	2/8 (25%)
- Bone marrow aplasia	1/193 (0.5%)	1/8 (12.5%)
- Evans’ syndrome	1/193 (0.5%)	1/8 (12.5%)
- Myelofibrosis	1/193 (0.5%)	1/8 (12.5%)
- Sickle cell anemia		
Degree of immunosuppression		
- Not immunosuppressed	49 (25.4%)	
- Mild immunosuppression	77 (39.9%)	
- Moderate immunosuppression	17 (8.8%)	
- Severe immunosuppression	50 (25.9%)	
Symptoms		
- No	23 (11.9%)	
- Yes	170 (88.1%)	
- Cough	54 (31.7%)	
- Dyspnea	71 (41.7%)	
- Fewer	129 (75.8%)	
Laboratory data at first evaluation	Mean ± SD	
- White blood count (in cells/µL)	6.5 ± 11.4	
- Neutrophils (in cells/µL)	4.1 ± 7.9	
- Lymphocytes (in cells/µL)	3.7 ± 21.3	
- Hemoglobin (in g/dL)	10.0 ± 2.2	
- Platelets (in units/µL)	149.7 ± 369.1	
- C-reactive protein (in mg/dL)	28.3 ± 60.8	
- Procalcitonin (in ng/mL)	0.3 ± 2.5	
- Creatinine (in mg/dL)	0.9 ± 0.4	
Laboratory data before bronchoscopy	Mean ± SD	
- White blood count (in cells/µL)	6.2 ± 12.9	
- Neutrophils (in cells/µL)	3.1 ± 5.1	
- Lymphocytes (in cells/µL)	1.0 ± 1.4	
- Hemoglobin (in g/dL)	10.1 ± 5.1	
- Platelets (in units/µL)	118.9 ± 130.5	
- C-reactive protein (in mg/dL)	30.4 ± 57.2	
- Procalcitonin (in ng/mL)	1.5 ± 4.2	
- Creatinine (in mg/dL)	0.8 ± 0.5	
HRCT findings		
- Negative	6/187 (3.2%)	
- Consolidations	56/187 (29.9%)	
- Ground glass opacities	67/187 (35.8%)	
- Nodular opacities	51/187 (27.2%)	
- Pleural effusion	42/187 (22.4%)	

Abbreviations: HRCT, high-resolution computed tomography; SD, standard deviation.

**Table 2:** Correlations between days from symptom onset and bronchoscopy with BAL and laboratory findings at first evaluation and close to BAL.

	Correlation Coefficient r (95% Confidence Interval)
At first evaluation in all patients	
White blood count (in cells/µL)	0.032 (−0.119 to 0.182), n.s.
Neutrophils (in cells/µL)	−0.065 (−0.228 to 0.101), n.s.
Lymphocytes (in cells/µL)	−0.038 (−0.182 to 0.107), n.s.
Hemoglobin (in g/dL)	0.229 (0.081 to 0.367), *p* = 0.002
Platelets (in units/µL)	0.091 (−0.059 to 0.237), n.s.
C-reactive protein (in mg/dL)	0.112 (−0.034 to 0.255), n.s.
Procalcitonin (in ng/mL)	0.017 (−0.322 to 0.352), n.s.
Creatinine (in mg/dL)	0.231 (0.091 to 0.364), *p* = 0.001
At first evaluation in inpatients	
White blood count (in cells/µL)	−0.046 (−0.213 to 0.123), n.s.
Neutrophils (in cells/µL)	−0.091 (−0.271 to 0.093), n.s.
Lymphocytes (in cells/µL)	0.023 (−0.142 to 0.188), n.s.
Hemoglobin (in g/dL)	0.099 (−0.070 to 0.263), n.s.
Platelets (in units/µL)	−0.128 (−0.290 to 0.039), n.s.
C-reactive protein (in mg/dL)	0.219 (0.055 to 0.372), *p* = 0.009
Procalcitonin (in ng/mL)	−0.023 (−0.386 to 0.346), n.s.
Creatinine (in mg/dL)	0.017 (−0.148 to 0.181), n.s.
**Close to the BAL**	
White blood count (in cells/µL)	−0.061 (−0.216 to 0.097), n.s.
Neutrophils (in cells/µL)	−0.012 (−0.185 to 0.160), n.s.
Lymphocytes (in cells/µL)	0.024 (0.072 to 0.410), *p* = 0.006
Hemoglobin (in g/dL)	0.108 (−0.049 to 0.260), n.s.
Platelets (in units/µL)	0.120 (−0.037 to 0.272), n.s.
C-reactive protein (in mg/dL)	0.118 (−0.030 to 0.261), n.s.
Procalcitonin (in ng/mL)	0.271 (−0.027 to 0.526), n.s.
Creatinine (in mg/dL)	0.184 (0.039 to 0.321), *p* = 0.01
**Close to the BAL in inpatients**	
White blood count (in cells/µL)	−0.084 (−0.253 to 0.089), n.s.
Neutrophils (in cells/µL)	−0.087 (−0.274 to 0.104), n.s.
Lymphocytes (in cells/µL)	0.363 (0.177 to 0.524), *p* = 0.0002
Hemoglobin (in g/dL)	0.049 (−0.123 to 0.219), n.s.
Platelets (in units/µL)	0.025 (−0.148 to 0.197), n.s.
C-reactive protein (in mg/dL)	0.229 (0.063 to 0.382), *p* = 0.007
Procalcitonin (in ng/mL)	0.302 (−0.002 to 0.555), n.s.
Creatinine (in mg/dL)	0.048 (−0.117 to 0.212), n.s.

Abbreviations: n.s., non-statistically significative.

**Table 3:** Microorganisms isolated and antimicrobial therapies.

	N (%)	
Number of BAL	193	
BAL positive of at least one microorganism	104 (53.9%)	
BAL negative	89 (46.1%)	
Number of microorganisms isolated	192	
Bacteria and mycobacteria	55/192 (28.6%)	
- *Pseudomonas aeruginosa*	9/192 (4.7%)	9/55 (16.3%)
- *Stenotrophomonas maltophilia*	7/192 (3.6%)	7/55 (12.7%)
- *Escherichia coli*	6/192 (3.1%)	6/55 (10.9%)
- *Actynomices* spp.	4/192 (2.1%)	4/55 (7.2%)
- *Stafiococcus aureus*	4/192 (2.1%)	4/55 (7.2%)
- *Acinetobacter baumannii*	4/192 (2.1%)	4/55 (7.2%)
- *Enterobacter cloacae*	3/192 (1.6%)	3/55 (5.4%)
- *Haemophilus influenzae*	2/192 (1%)	2/55 (3.6%)
- *Legionella pneumophila*	2/192 (1%)	2/55 (3.6%)
- *Klebsiella pneumoniae*	2/192 (1%)	2/55 (3.6%)
- *Nocardia* spp.	2/192 (1%)	2/55 (3.6%)
- *Veilonella*	1/192 (0.5%)	1/55 (1.8%)
- *Mycoplasma pneumoniae*	1/192 (0.5%)	1/55 (1.8%)
- *Fusobacterium*	1/192 (0.5%)	1/55 (1.8%)
- *Moraxella catarrhalis*	1/192 (0.5%)	1/55 (1.8%)
- *Straptococcus constellatus*	1/192 (0.5%)	1/55 (1.8%)
- *Elizabethkingia mirincola*	1/192 (0.5%)	1/55 (1.8%)
- *Streptococcus pneumoniae*	1/192 (0.5%)	1/55 (1.8%)
- *Mycobacterium tuberculosis*	2/192 (1%)	2/55 (3.6%)
- *Mycobacterium xenopi*	1/192 (0.5%)	1/55 (1.8%)
Viruses	114/192 (59.4%)	
- HHV-6	30/192 (15.6%)	30/114 (26.3%)
- EBV	27/192 (14.1%)	27/114 (23.7%)
- CMV	25/192 (13%)	25/114 (21.9%)
- Rhinovirus	8/192 (4.2%)	8/114 (7%)
- HSV-1	7/192 (3.6%)	7/114 (6.1%)
- RSV	4/192 (2.1%)	4/114 (3.5%)
- SARS-CoV-2	4/192 (2.1%)	4/114 (3.5%)
- Metapneuovirus	2/192 (1%)	2/114 (1.8%)
- Adenovirus	2/192 (1%)	2/114 (1.8%)
- Influenza A	1/192 (0.5%)	1/114 (0.9%)
- Parainfluenza 3	1/192 (0.5%)	1/114 (0.9%)
- HHV-7	1/192 (0.5%)	1/114 (0.9%)
- BKV	1/192 (0.5%)	1/114 (0.9%)
- H3N2	1/192 (0.5%)	1/114 (0.9%)
Fungi	23/192 (12%)	
- *Pneumocystis jirovecii*	10/192 (5.2%)	10/23 (43.5%)
- *Aspergillus* spp.	7/192 (3.6%)	7/23 (30.4%)
- *Candida* spp.	5/192 (2.6%)	5/23 (21.7%)
- *Fusarium* spp.	1/192 (0.5%)	1/23 (4.4%)
Prophylaxis with antibiotics	137/150 (91.3%)	
- TMP/SMX	29/137 (21.2%)	
- TMP/SMX + Aciclovir	86/137 (62.7%)	
- TMP/SMX + Valaciclovir	22/137 (16.1%)	
Prophylaxis with antivirals	13/150 (8.7%)	
- Aciclovir	7/150 (4.7%)	
- Valaciclovir	6/150 (4%)	
BAL performed in course of:	Available data in 190 cases	
- Antibiotics	138/190 (72.6%)	
- Antibiotic prophylaxis	88/190 (46.3%)	
- Antiviral prophylaxis	27/190 (14.2%)	
Number of antimicrobial drugs ongoing when BAL was performed	Available data in 112 cases	
- 0	14/112 (12.5%)	
- 1	14/112 (12.5%)	
- 2	35/112 (31.3%)	
- 3	24/112 (21.4%)	
- 4	22/112 (19.6%)	
- >4	3/112 (2.7%)	

Abbreviation list: CMV, Cytomegalovirus; EBV, Epstein–Barr virus; HHV-6, Human herpes virus-6; HHV-7, Human herpes virus-7; HSV-1, Herpes simplex virus-1; RSV, Respiratory syncytial virus; SARS-CoV-2, Severe Acute Respiratory Syndrome Coronavirus 2; TMP/SMX, Trimethoprim/sulfamethoxazole.

**Table 4:** Survival and BAL isolations: number of patients alive at days 0, 10, 20, and 30.

	Days from Symptom Onset				*p* Value
	0	10	20	30	
Bacterium pos	32	32	29	29	0.53
Bacterium neg	110	110	106	103	
Virus pos	60	60	56	54	0.12
Virus neg	82	82	79	78	
Fungi pos	19	19	17	16	0.29
Fungi neg	123	123	118	116	
	**Days from BAL**				
Bacterium pos	32	29	27	25	0.24
Bacterium neg	110	107	103	99	
Virus pos	60	57	53	50	0.09
Virus neg	82	79	77	74	
Fungi pos	19	17	16	14	0.19
Fungi neg	123	119	114	110	

## Data Availability

Data available upon request due to restrictions (privacy).
